# Rescue Therapy With Pegcetacoplan in a Patient With Proliferative Glomerulonephritis With Monoclonal Immunoglobulin Deposits

**DOI:** 10.1016/j.ekir.2026.103770

**Published:** 2026-01-08

**Authors:** Hormaz Dastoor, Emad Khater, Suhail Al Salam, Stephen G. Holt

**Affiliations:** 1 Departement of Nephrology, SEHA - Kidney Care, SEHA Abu Dhabi Health Services Ltd, Abu Dhabi, United Arab Emirates; 2 Department of Pathology, College of Medicine and Health Sciences, United Arab Emirates University, Al Ain, United Arab Emirates; 3 Departement of Nephrology, Khalifa University, Abu Dhabi, United Arab Emirates

To the Editor:

In the pivotal phase 2 and phase 3 VALIANT trials, pegcetacoplan reduced proteinuria and stabilised kidney function in patients with C3 glomerulopathy and primary immune complex membranoproliferative glomerulonephritis.^1^^,^^2^ However, the trials enrolled patients with an estimated glomerular filtration rate (eGFR) ≥ 30 ml/min per 1.73 m^2^,^2^ leaving limited evidence in this population. We report the successful use of pegcetacoplan in a patient with severe biopsy-proven proliferative glomerulonephritis with monoclonal immunoglobulin deposits and low eGFR with impending dialysis.

A 50-year-old man with diet-controlled type 2 diabetes, gout, and nephrolithiasis presented with vomiting. Laboratory tests showed serum creatinine of 240 μmol/l, urine protein-to-creatinine ratio (uPCR) of 1.9 g/g, and microscopic haematuria. Serological workup, including complement levels (C3, C4), serum and urine electrophoresis, immunofixation electrophoresis, and peripheral blood flow cytometry were all negative or within reference range.

Renal biopsy revealed mesangial and endocapillary hypercellularity with glomerular basement-membrane duplication, consistent with an membranoproliferative glomerulonephritis pattern (Supplementary Figure S1). Periodic acid–Schiff and silver methenamine staining confirmed mesangial expansion and basement-membrane remodelling. Immunofluorescence demonstrated C3 (2+), IgG (2+), C1q (2+), and kappa light-chain (2+) deposition, with negative lambda staining (Supplementary Figure S2). Secondary causes were excluded, suggesting a diagnosis of proliferative glomerulonephritis with monoclonal immunoglobulin deposits.^3^ About 25% of glomeruli showed global sclerosis, and 25% tubulointerstitial fibrosis.

The patient received corticosteroids, mycophenolate mofetil, and renin-angiotensin system inhibitors, achieving partial improvement (creatinine: 178 μmol/l, eGFR: 38 ml/min per 1.73 m^2^, uPCR: 0.92 g/g). Repeat biopsy after 5 months showed reduced C3 intensity but persistent IgG, C1q, and kappa staining. Despite therapy and a later addition of a sodium-glucose cotransporter-2 inhibitor, renal function worsened (creatinine: 417 μmol/l, eGFR: 13 ml/min per 1.73 m^2^, uPCR: 5 g/g).

Pegcetacoplan was initiated as rescue therapy, dosed as subcutaneous infusions of 1080 mg twice a week, after prophylactic vaccinations against encapsulated organisms. Within 6 weeks, serum creatinine improved to 196 μmol/l (eGFR: 34 ml/min per 1.73 m^2^) and uPCR declined to 0.86 to 1.6 g/g. After 9 months, creatinine was 160 μmol/l, eGFR was 42 ml/min per 1.73 m^2^ and uPCR 1.5g/g (Figure 1). Complement levels increased (C3: 4.45 g/l; C4: 0.42 g/l). Treatment was well-tolerated without any infections or side effects.

**Figure 1:**
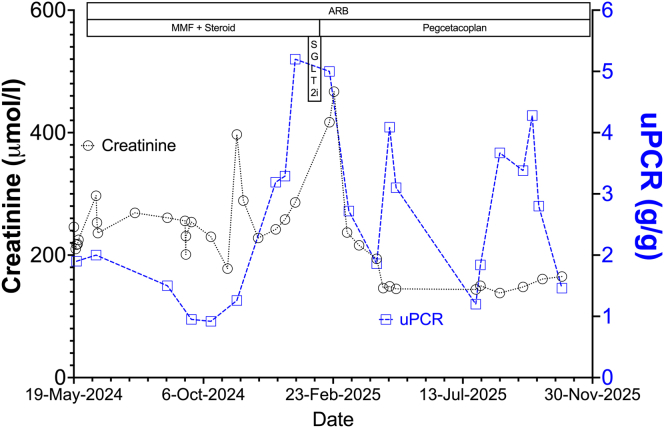
Temporal trends in kidney function. Initial therapy with steroids and mycophenolate mofetil led to transient improvement, followed by progressive deterioration. Sodium-glucose cotransporter-2 inhibitor was introduced in January 2025 as a last-line option but failed to halt disease progression. After pegcetacoplan initiation in late February 2025, a marked and sustained improvement in renal function was observed, with serum creatinine declining, eGFR increasing. and a slow improvement in proteinuria.

This case illustrates the real-world effectiveness of pegcetacoplan in a patient with biopsy-proven proliferative glomerulonephritis with monoclonal immunoglobulin deposits who underwent rescue therapy after failure of initial immunosuppressive therapy. Future studies are warranted to evaluate the safety, timing, and long-term outcomes of pegcetacoplan in broader and more advanced real-world populations with proliferative glomerulonephritis with monoclonal immunoglobulin deposits, as an alternative to more toxic therapies with plasma cell and lymphocyte cell directed clone therapies.

## Disclosure

All the authors declared no competing interests.

## Patient Consent

Written informed consent has been obtained from the patient to publish this paper.

## Supplementary Material

Supplementary File (PDF) **Figure S1.** Native kidney biopsy. **Figure S2.** Immunofluorescence staining of native kidney biopsy.

## Footnotes

Supplementary File (PDF)
**Figure S1.** Native kidney biopsy. **Figure S2.** Immunofluorescence staining of native kidney biopsy.
