# Supplementary material for: Rescue Therapy With Pegcetacoplan in a Patient With Proliferative Glomerulonephritis With Monoclonal Immunoglobulin Deposits
Source: Kidney Int Rep. 2026 Jan 8;11(3):103770. doi: 10.1016/j.ekir.2026.103770 (PMC12860243; doi:10.1016/j.ekir.2026.103770)
Supplement: Supplementary File (PDF) — Figure S1. Native kidney biopsy. Figure S2. Immunofluorescence staining of native kidney biopsy. [file mmc1.pdf]

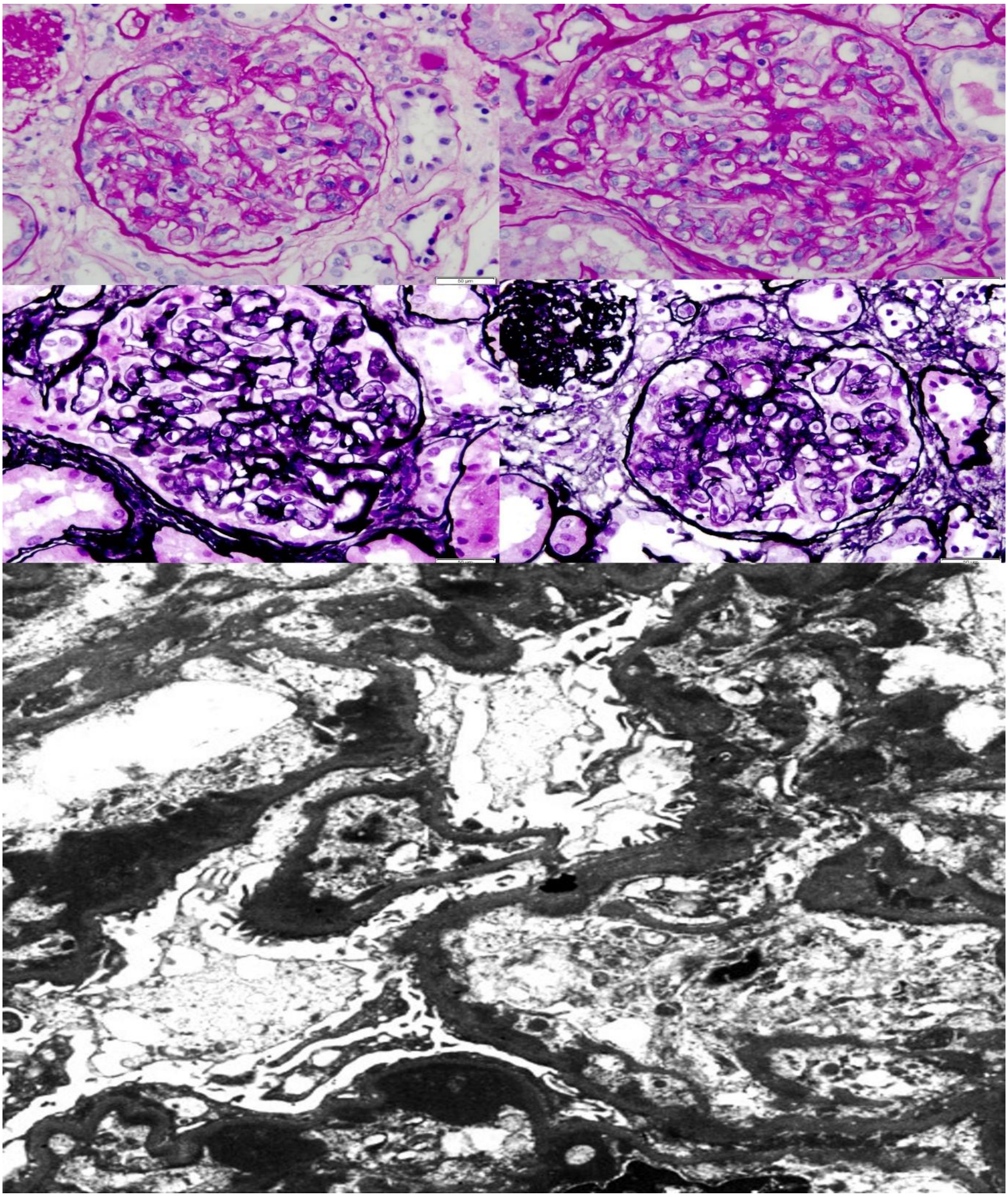

Supplementary Figure S1: Native kidney biopsy. Periodic acid–Schiff (PAS) staining shows mesangial and endocapillary hypercellularity with segmental duplication of the glomerular basement membrane. Silver methenamine staining highlights glomerular basement membrane remodelling with double contours and capillary wall thickening. (e) Electron microscopy reveals subendothelial and mesangial electron-dense deposits, consistent with C3-dominant glomerulonephritis (PDF).

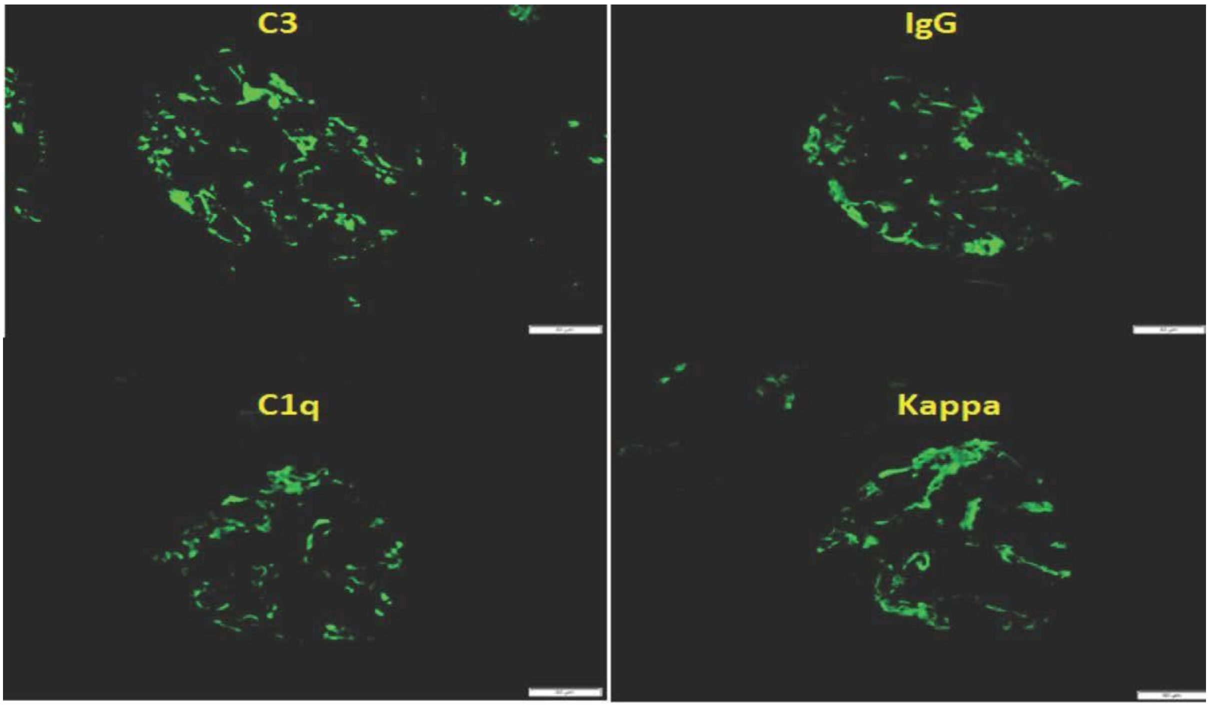

Supplementary Figure S2: Immunofluorescence staining of native kidney biopsy. Immunofluorescence microscopy shows dominant C3 deposition (3+) in a granular pattern along the glomerular capillary walls and mesangium. Additional staining is observed for IgG (2+), C1q (2+), and kappa light chains (2+), while lambda light chains were negative (not shown). The staining pattern supports a diagnosis of C3-dominant glomerulonephritis, with consideration of a possible underlying monoclonal gammopathy, subsequently ruled out by normal serum and urine electrophoresis and light chain assays (PDF).
